# Safeguarding Society from the Unfit

**Published:** 1911-02

**Authors:** A. B. Cooke

**Affiliations:** Nashville, Tenn.


### Safeguarding Society from the Unfit.

BY A. B. COOKE, M. D., NASHVILLE, TENN.

The science of medicine is not concerned with abstractions. Whether heredity or environment is the most potent factor in determining the social value of an individual is of little real moment. The practical consideration concerning which there is no dispute, is that the characteristics and tendencies, mental and physical, of the parent appear with striking uniformity in the offspring. This is conspicuously true of the class known as degenerates. No observing man—more particularly no observing physician—can have failed to note in his own experience many instances in which abnormal traits or vicious tendencies have been faithfully reproduced in one or all the children. The stock raiser recognizes this principle when he places at the head of his flock or herd only animals possessing the most desirable qualities. However "classy" in appearance a stallion may be, if he is ugly tempered or shows defects in gait or action, his services in the stud are not in demand. The dog fancier jealously guards his kennel from contamination by impure and alien strains. And even in regard to the humbler forms of life of the vegetable world, attention is constantly directed to the maintenance and improvement of the standard of quality.

Yet with respect to human beings such considerations are totally disregarded. The mentally defective, the morally abandoned, the physically unfit are permitted to display their procreative prowess without let or hindrance, while society patiently and calmly reaps the inevitable harvest. Viewed from the pecuniary side the cost of the degenerate classes, as represented by prisons, asylums and kindred institutions, is greater to the nation than the aggregate cost of its army and navy; from the standpoint of suffering and death, the cost doubtless exceeds that of war and pestilence combined.

With the exception of a few progressive States, no attempt is being made to protect the public from this enormous economic loss and practically no concerted effort along humanitarian lines in the interest of the unfortunates themselves. Of course, the State must provide for the helpless, but in such provision the welfare of society is not less to be considered than the interests of the individual. The punitive idea which dominates our prison system is an anachronism in our modern civilization. If criminals are such by reason of conditions which they had no voice in shaping and which they are powerless to modify or control, the chief purpose of the State should be to afford them every assistance possible, at the same time that the larger interests of society are being safeguarded. But in spite of the well-intentioned efforts of social reformers and the adoption of more humane methods of management by the majority of States, little tangible result is achieved along the lines indicated. On the contrary, the average inmate of penitentiary or reformatory emerges hardened and embittered against society, and, sad to say, with a larger knowledge of evil than when he entered. Fear of a prison sentence is not an adequate deterrent from crime. The constantly overcrowded condition of our penal institutions is due chiefly to the presence of habitual transgressors. A single conviction is the exception rather than the rule. Many instances can of course be cited of a first offense and terms of service being followed by complete and permanent reformation; but in the light of prison records the chances in a given case are all in favor of a second or even a third conviction. Prisons, then, are not effective as preventives of crime, and, it may be observed in passing, capital punishment is little more so. The penalty of murder or rape is death, yet murders and rapes are daily being committed by those who have a full knowledge of the probable consequences.

The calendars of our criminal courts are more congested today than at any time in our country's history, and the proportion of recognized criminals to the general population, as shown by the census reports, is constantly on the increase.

The following figures are official:

In 1850 there were in the United States 6737 prisoners, the ratio of population being 1 in 3442; in 1860 there were 19,086 prisoners, ratio to population, 1 in 1647; in 1870 there were 32,901 prisoners, ratio to population, 1 in 1171; in 1880 there were 58,608 prisoners, ratio to population, 1 in 855; in 1890 there were 82,329 prisoners, ratio to population, 1 in 757; in 1900 there were 133,280 prisoners, ratio to population, 1 in 586; in the present census year the ratio will almost certainly figure out 1 in less than 500.*

Glancing for a moment at the official records for Tennessee, which may be taken as at least fairly representing the conditions prevailing in all of the Southern States, we find that, during the biennial period 1898-1900, the number of new convicts received was 1212; for the period 1902-4 there were 1267; for the period 1906-8 there were 1327; for the year 1909 there were 810, or, if this rate is maintained for the two-year period 1908-10, the figures will be 1620.

The records further show that on December 1, 1900, the total number of State convicts on hand was 1516; on December 1, 1904, there were 1639; on December 1, 1908, there were 1775; on December 1, 1909, there were 1812.

Comparing these figures for the years 1900 and 1909, we find that the number of new convicts increased 34 per cent in the nine years, and that the total number of convicts on hand increased nearly 20 per cent.

It is hardly reasonable to suppose that the general population of Tennessee increased 34 per cent in this nine-year period and we are therefore forced to conclude that both actually and relatively crime is steadily increasing in the State.

Only one interpretation of these figures is possible, namely, that there is some vital defect, either of omission or of commission, with reference to our system of criminal laws, and it would seem that the time has fully come when any amendment or addition to them which promises to better conditions should be given a fair trial. It is the consensus of opinion of social reformers and criminologists that the only effective way to correct the evil is by restricting propagation among the criminal and degenerate classes. What is known as the "Indiana Movement" is based upon this idea. Two laws have been enacted and are in operation in that enlightened Commonwealth, which already foreshadow the solution of the vexed problem.

The first of these, passed in 1905, concerns the regulation of marriage:

"No license to marry shall be issued except upon written and verified application. The form of application shall be supplied by the State Board of Health, and said Board may revise said form from time to time, as may be advisable. No license to marry shall be issued when either of the contracting parties is an

*"Hereditary Criminals," by Chas. V. Carrington, Virginia *Medical Semi-Monthly*, April 8, 1910.

imbecile, epileptic, of unsound mind or under guardianship as a person of unsound mind, nor to any male person who is or has been, within the last five years, an inmate of any county asylum, or home for indigent persons; nor shall any license issue when either of the contracting parties is afflicted with a transmissible disease."

A marriage in violation of this law is declared to be illegal, and any county court clerk who issues a license contrary to law and any person performing the ceremony when the applicants have no license are subject to a fine of $100.

But recognizing that this law, even though stringently enforced, would necessarily be slow and imperfect in operation, a second law aiming more directly at the evil was enacted in 1907, as follows:

"Whereas, Heredity plays a most important part in the transmission of crime, idiocy and imbecility; therefore,

Be it enacted by the General Assembly of the State of Indiana, That on and after the passage of this act it shall be compulsory for each and every institution in the State entrusted with the care of confirmed criminals, idiots, rapists and imbeciles to appoint upon its staff, in addition to the regular institutional physician, two (2) skilled surgeons of recognized ability, whose duty it shall be, in conjunction with the chief physician of the institution, to examine the mental and physical condition of such inmates as are recommended by the institutional physician and Board of Managers.

If, in the judgment of this committee of experts and the Board of Managers, precreation is inadvisable and there is no probability of improvement of the mental and physical condition of the inmates, it shall be lawful for the surgeon to perform such operation for the prevention of procreation as shall be decided safest and most effective. But this operation shall not be performed except in cases that have been pronounced unimprovable.

*Provided,* That in no case shall the consultation fee be more than three ($3.00) dollars to each expert, to be paid out of the funds appropriated for the maintenance of such institution."

The father and champion of this latter law was Dr. H. C. Sharp, of Indianapolis, who as surgeon of the Indiana Reformatomy at Jeffersonville carried out its provisions in 412 cases during the first year after its passage. Previous to that time he had operated in the same institution upon 310 prisoners who voluntarily submitted.

The operation which Dr. Sharp has employed and popularized is that of double vasectomy. It is exceedingly simple in technic, is very quickly done, requires little or no loss of time on the part of the subject, is readily performed under local anesthesia, and is practically free from danger. It is performed as follows: Under strict aseptic precautions, the vas is grasped between the thumb and forefinger of the left hand at the site selected and a few drops of anesthetic solution injected into the overlying scrotal tissues. A small incision is then made and the vas drawn through, cleared of all other structures, ligated and severed below the ligature. A half-inch section of the end next to the testicle is removed, but not ligated, so as not to impede the escape of the testicular secretion. The wound is then closed with a single suture and a suspensory or other form of support applied. There is said to be no shock and the patient resumes his accustomed occupation within a few hours. Performed in this way there is no atrophy or other pathologic change in the testicle, no diminution of sexual power or pleasure, and no impairment of the general health. On the other hand a change is soon noticed in these patients, may of whom are sexual perverts, due to the fact that the secretion is diverted from the seminal vesicles, and their chronic tension and irritability relieved. All observers agree that vicious practices are quickly lessened or abandoned, and the entire nature of the subject, mental, moral and physical, greatly improved. So pronounced is the benefit that the patient himself soon realizes it and is not only grateful, but seeks to induce his fellows to undergo the same treatment.

As regards the female, the application of the provisions of the law is a more serious matter. But in this day of perfected surgical technic ligation or resection of the Fallopian tubes is not a formidable procedure and the results which follow are no less satisfactory and desirable.

Vasectomy is not specified as the method of sterilization in the Indiana law, but its advantages over other methods are obvious. Orchidectomy is a mutilating operation and one of considerable gravity, yet I am persuaded that it should be recognized and specified as the only method permissible in a certain class of criminals. I refer to rapists. Human nature will never rise superior to the idea that this type of criminal merits *punishment,* and punishment of a character which will render a repetition of the crime forever impossible. As a preventive, particularly in the South, where this monstrous evil is most prevalent and most abhorrent, it can not be doubted that the legal infliction of one such penalty would have a more salutary effect than many lynchings or even burnings at the stake.

The possibility of sterilization by the X-rays deserves a word of mention, in this connection. The danger of such a result even in the therapeutic application of the agent is universally recognized and guarded against; but the questions of permanency and dependability remain to be settled. In the case of the male this method may prove to be entirely feasible and, if so, will offer many advantages over surgical measures.

The foregoing remarks have been in the main directed to habitual criminals. But they are just as pertinent and just as applicable to the larger class comprehended under the general term "defectives" or "degenerates." Unrestrained by any sense of responsibility, the hopelessly insane, the congenital idiot, the confirmed inebriate, the imbecile and the sexual pervert embrace every opportunity to gratify their animal instincts without thought of consequences. Left to pursue their own bent, a horde of similar defectives is sure to result. It is a startling thought that the birth rate of this class is increasing, as shown by statistics, fully twice as fast as that of responsible, law-abiding citizens. There is no reason either in justice or mercy why this state of things should be indefinitely tolerated. The law of self-preservation applies to society equally as to the individual, and mere sentimental considerations should have no weight in the face of the issues at stake. The ever-present necessity for new institutions, or additions to old ones, for the care of this class of public charges, emphasizes the importance and urgency of the question. Society has the right, and should exercise it, to protect itself from this constantly increasing burden, especially when the means at hand is as safe and humane as it would certainly be effective.

Apropos of the general subject, the following appalling facts, stated in the form of questions, from a recent issue of *The World Today*, are both pertinent and illuminating:

"Do you know that our criminals cost us $3,500,000 a day?

"Do you know that 250,000 persons—whom the law never touches—are engaged in the systematic pursuit of crime as a business?

"Do you know that the pickpockets of New York retain the permanent services of one of the best known lawyers in the United States to look after their 'interests'?

"Do you know that during the past ten years the tramp burglars of this country have almost doubled in number?

"Do you know that this army of criminals is partly made up of mental defectives who might have been placed, as children, in institutions where they would have been trained to lead harmless and, perhaps, useful lives?"

Let me add a few questions to this list:

Do you know that not a single public institution exists in Tennessee or any other Southern State for the care and training of defective children?

Do you know that if sterilization of the unfit had been adequately employed during the past generation no such history of crime and degeneracy as that above set forth would be true today?

Do you know that if this remedy were applied with discriminating thoroughness *now,* the future welfare of society would be more effectively safeguarded than by any other means, and at the same time many an unpromising life rescued and unlifted?

Do you not believe that the appeal of posterity for a purer and cleaner social structure should be heard and heeded?

Do you realize that no more vital subject than this could engage the attention of this body?

That the importance of this great question is coming to be generally recognized is evidenced in the fact that such widely separated States as Kansas, Delaware, Indiana, Connecticut, Michigan and New Jersey have enacted laws regulating marriage, while Utah, Oregon, California, Connecticut and possibly others have followed Indiana's lead in legalizing the sterilization of criminals and degenerates.

It is yet too early to point to definite results from the operation of these laws. But when the crucial test of time shall have been applied I believe it is reasonable to predict that crime and degeneracy will be largely decreased and the world be correspondingly better and happier.

The Southern Medical Association should lead in all matters looking to the welfare of the Southland, which it loves and serves. If it were not beyond the proper province and propriety of this paper, I should close it by offering a resolution formally endorsing the movement herein discussed, and commending it to the active interest and effort of the several component State societies.—*Southern Medical Journal.*

